# Programmed cell death in host-symbiont associations, viewed through the Gene Ontology

**DOI:** 10.1186/1471-2180-9-S1-S5

**Published:** 2009-02-19

**Authors:** Marcus C Chibucos, Candace W Collmer, Trudy Torto-Alalibo, Michelle Gwinn-Giglio, Magdalen Lindeberg, Donghui Li, Brett M Tyler

**Affiliations:** 1 Virginia Bioinformatics Institute, Virginia Polytechnic Institute and State University, Blacksburg, VA 24061, USA; 2 Current address: Institute for Genome Sciences, University of Maryland School of Medicine, Baltimore, MD 21201, USA; 3 Department of Biological and Chemical Sciences, Wells College, Aurora, NY 13026, USA; 4 Department of Plant Pathology and Plant-Microbe Biology, Cornell University, Ithaca, NY 14853, USA; 5 The *Arabidopsis *Information Resource, Carnegie Institution, Department of Plant Biology, Stanford CA 94305, USA

## Abstract

Manipulation of programmed cell death (PCD) is central to many host microbe interactions. Both plant and animal cells use PCD as a powerful weapon against biotrophic pathogens, including viruses, which draw their nutrition from living tissue. Thus, diverse biotrophic pathogens have evolved many mechanisms to suppress programmed cell death, and mutualistic and commensal microbes may employ similar mechanisms. Necrotrophic pathogens derive their nutrition from dead tissue, and many produce toxins specifically to trigger programmed cell death in their hosts. Hemibiotrophic pathogens manipulate PCD in a most exquisite way, suppressing PCD during the biotrophic phase and stimulating it during the necrotrophic phase. This mini-review will summarize the mechanisms that have evolved in diverse microbes and hosts for controlling PCD and the Gene Ontology terms developed by the Plant-Associated Microbe Gene Ontology (PAMGO) Consortium for describing those mechanisms.

## Introduction

Programmed cell death (PCD) is defined in the Gene Ontology (GO) as "GO: 0012501 cell death resulting from activation of endogenous cellular processes" [1]. PCD is a critical component of defense in both plants and animals against microbes, especially biotrophic pathogens that draw their nutrition from living tissue (reviewed in [2] and in this supplement [3]). Many developmental processes also rely upon PCD [4]. In vascular plants these include xylem vessel differentiation [5], autumnal leaf senescence [6], and development of root cap and mucilage cells [7]. In higher vertebrates these processes include digit formation and nervous system cell culling [8]. The role of PCD in the response to biotic stress, for plants in particular, has been reviewed many times elsewhere [6,9-11]. This review will focus on the struggle for control of PCD that occurs between diverse microbes and their plant and animal hosts, as well as the GO terms that have been developed recently by the Plant-Associated Microbe Gene Ontology (PAMGO) Consortium [12] to describe the processes underlying this struggle.

## The Gene Ontology

The GO is a controlled vocabulary comprised of GO terms that describe gene product attributes in any organism [13]. GO terms are arranged as directed acyclic graphs (DAGs) within three ontologies, "GO: 0005575 cellular component", "GO: 0008150 biological process", and "GO: 0003674 molecular function". DAGs differ from hierarchies in that each more specialized term (child) can be related to greater than one less specific term (parent). Multiple child terms (siblings) that share a common parent term are distinct, and yet they possess the common attributes of the parent, as what is true of a parent term must be true of any child term. Relationships among parent and child terms within a DAG are symbolized by arrows that reflect GO "is_a", "part_of", and "regulates" relationships; for example, "GO: 0001906 cell killing" is a type of "GO: 0008150 biological process", and thus these terms would be connected by the "is_a" relationship (for more information on term-term relationships and ontology structure, see [13]).

## Forms of cell death

### Programmed cell death

**Figure 1:**
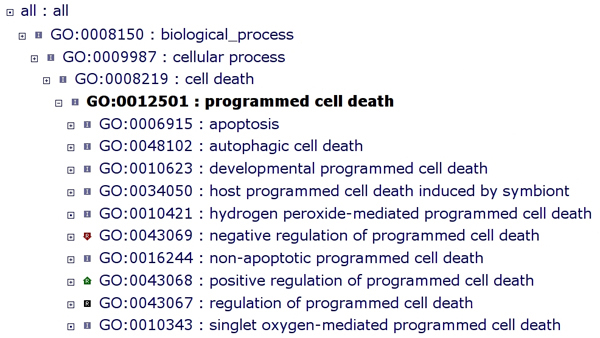
**"GO: 0012501 programmed cell death" and its child terms depicted in a screenshot of the Gene Ontology AmiGO browser **[1]. Most terms shown here below "GO: 0012501 programmed cell death" are types of programmed cell death, symbolized by the logo showing an "I" inside a square, which denotes the "is_a" relationship. However, three terms (various logos with "R") describe the "regulates" type of relationship. For more information on ontology structure, including term-term relationships, see [13].

Some of the major classes of PCD, as defined by the biological process ontology of GO, include "GO: 0006915 apoptosis" (sometimes called type I PCD), "GO: 0016244 non-apoptotic programmed cell death" (sometimes called type II PCD), "GO: 0048102 autophagic cell death", "GO: 0010623 developmental programmed cell death", and "GO: 0034050 host programmed cell death induced by symbiont"; "GO: 0009626 plant-type hypersensitive response" is a child term of "GO: 0034050 host programmed cell death induced by symbiont". In addition to these types of PCD, the GO differentiates two others (siblings of those above): "GO: 0010421 hydrogen peroxide-mediated programmed cell death" and "GO: 0010343 singlet oxygen-mediated programmed cell death" [1]. Figure 1 shows a screenshot of the AmiGO ontology browser at the Gene Ontology depicting "GO: 0012501 programmed cell death" and its child terms [1]. In addition to the terms describing classes of PCD, the GO contains three other terms, also shown in Figure 1, that describe types of PCD regulation: "GO: 0043067 regulation of programmed cell death", "GO: 0043069 negative regulation of programmed cell death", and "GO: 0043068 positive regulation of programmed cell death". Taken together, these terms describing both classes of PCD and regulation of PCD allow for annotations that capture various aspects of PCD as a biological process.

### Apoptosis and necrosis

Several types of PCD related to defense have been distinguished in the literature, for example apoptosis and the hypersensitive response (HR). Autophagy, a highly conserved PCD pathway related to protein and organelle turnover, also has been implicated in plant innate immunity (reviewed in [14]). Another commonly used but poorly defined term, "necrosis", is not included as a term in the GO because it is a phenotype, i.e. post-mortem observation of dead cells, not a process, and the GO does not include terms for describing phenotypes. Necrosis indicates that cell death has occurred, but not necessarily the process by which it was achieved [15]. There may be some cases where necrosis proceeds as a programmed process, but this is still poorly understood (see Note added in proof). Necrosis exists in the GO only as a synonym of the terms "GO: 0008219 cell death", "GO: 0001906 cell killing", "GO: 0019835 cytolysis", and "GO: 0012501 programmed cell death", but its use in describing a process is discouraged without great caution whether or not one is using GO. Similarly, use of the phrase "necrotic tissue" is discouraged in describing the results of cell death.

"GO: 0006915 apoptosis", on the other hand, exists in the GO as it constitutes a well-defined process. Apoptosis includes condensation of chromatin at the nuclear periphery, condensation and vacuolization of the cytoplasm and plasma membrane blebbing, followed by breakdown of the nucleus and fragmentation of the cell to form apoptotic bodies. Other characteristics of apoptosis include DNA fragmentation and the exposure of phosphatidyl serine on the cell surface [1,16]. The current GO definition of apoptosis is: "A form of PCD induced by external or internal signals that trigger the activity of proteolytic caspases, whose actions dismantle the cell and result in cell death. Apoptosis begins internally with the condensation and subsequent fragmentation of the cell nucleus (blebbing) while the plasma membrane remains intact..." [16]. As is true of all GO terms, it is likely that this definition will evolve as our understanding of apoptosis advances. Apoptosis frequently but inaccurately has been used as a synonym of PCD in the literature, creating confusion. This may be in part because apoptosis is also known as type I programmed cell death, but caution must be exercised to avoid inaccurate synonymous usage [15,17]. In the GO it is placed as a child term of "GO: 0012501 programmed cell death", reflecting the fact that it is considered a type of PCD.

### The hypersensitive response (HR)

Plants possess both a basal immune system, which recognizes microbe-associated molecular patterns (MAMPs, sometimes called PAMPs in the context of pathogens), and resistance gene (*R*-gene)-encoded proteins that can recognize pathogen gene products (reviewed in [18]), resulting in the activation of defenses. One form of plant defense is known as the hypersensitive response (HR). During the HR, reactive oxygen intermediates [19] and ion fluxes (Ca^2+ ^in particular [20]) lead to cell death, which is associated with defense activation and restriction of the pathogen [21,22]. The HR also initiates complex intracellular signalling that leads to transcription of defense genes [23]. HR is described in the GO as "GO: 0009626 plant-type hypersensitive response" and defined as "the rapid, localized death of plant cells in response to invasion by a pathogen" [1].

There are many parallels between plant-type HR and animal apoptosis, including the common features of chromatin condensation, activation of cysteine proteases, cytochrome *c *release, loss of membrane potential delta psi, and cytoplasmic shrinkage (reviewed in [4,24,25]). Yet there are significant differences. ATP dependence, nuclear shrinking, and engulfment by neighbouring cells are associated with animal apoptosis but not with plant HR. Vacuolization and mitochondrial swelling occur in plant HR but not animal apoptosis. Furthermore, DNA laddering, a common feature of animal apoptosis, is not always observed in plants [4,24]. Despite these differences, it is clear that diverse groups of host organisms use largely similar approaches to halt the spread of infectious pathogens.

Precisely distinguishing among the various modes of cell death remains an active ongoing topic [26-28], as does assigning corresponding GO terms to those modes. A great deal of recent work has focused on the molecular mechanisms underlying various kinds of cell death [29], including mitochondrial fusion and fission machinery [30]. Additional file 1 displays some common concepts related to endogenous cell death, i.e. cell death within an organism controlled by that organism itself, as well as associated GO terms created to describe those phenomena, with definitions and comments (depicted in greater detail than in Figure 1). Three of the GO terms shown in the table have comments suggesting alternative GO terms to use for annotating gene products related to host-symbiont interactions. PCD as it relates to host-symbiont interactions is discussed throughout the remainder of this review.

## PCD and host-symbiont interactions

A critical consideration regarding annotation of PCD-related gene products is whether PCD (including triggering or inhibition of PCD) is self-originating or extrinsically influenced, as may occur in symbiotic interactions. Note that in the GO, "symbiosis" comprises all symbiotic relationships between species along a continuum from mutualism through parasitism; "symbiont" and "host" are defined as the smaller and larger of the organisms, respectively, involved in a symbiotic interaction [12] (see "GO: 0044403 symbiosis, encompassing mutualism through parasitism" [1] for more information). Because the manipulation of PCD in one organism by a second organism during symbiotic interaction is extrinsic in nature, the PAMGO Consortium developed a new set of GO terms to describe processes related to extrinsic manipulation of PCD. These terms are for annotation of gene products produced by one organism that affect PCD in a second organism, and they are distinct from the previously existing GO terms appropriate for annotating genes involved in the purely endogenous processes within a single organism. For example, the GO definition of "GO: 0012501 programmed cell death" carries the comment: "...this term should be used to annotate gene products in the organism undergoing the programmed cell death. To annotate genes in another organism whose products modulate programmed cell death in a host organism, consider the term 'modulation by symbiont of host programmed cell death; GO:0052040'" [1] (Additional file 1). Similarly, the GO term "GO: 0009626 plant-type hypersensitive response" carries the comment "...this term is to be used to annotate gene products in the plant. To annotate symbiont gene products that induce the hypersensitive response, consider the biological process term 'modulation by symbiont of host defense-related programmed cell death; GO:0034053'" [1] (Additional file 1).

**Figure 2:**
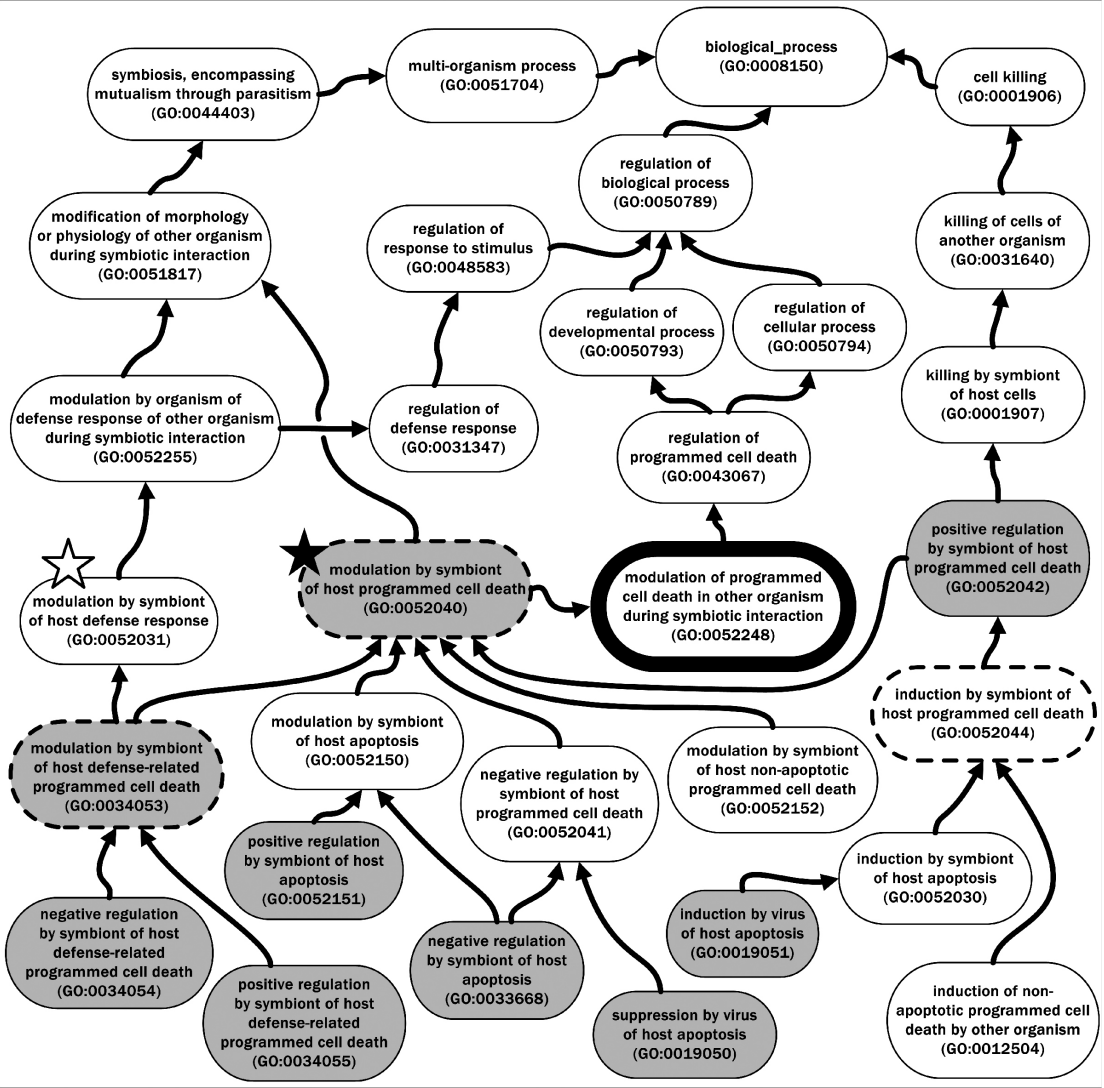
**Selected GO terms related to "GO: 0052040 modulation by symbiont of host programmed cell death"**. A greatly simplified directed acyclic graph (DAG) showing key low-level terms describing modulation of programmed cell death in one organism (the host) by another organism (the symbiont) is depicted. A simplified lineage for these terms is shown up to "GO: 0008150 biological_process". Only selected terms are shown, and only a few of the parent-child relationships are depicted; arrows symbolize GO "is_a" and "part_of" relationships (for more information on ontology structure, i.e. "is_a", "part_of", and "regulates", see [13]). Note that "GO: 0052040 modulation by symbiont of host programmed cell death" (denoted by a dark star) and "GO: 0052031 modulation by symbiont of host defense response" (light star) both ultimately exist under the "GO: 0051704 multi-organism process" node. The GO terms shaded with grey represent annotations discussed in the text; GO terms highlighted with broken lines or black serve as reference points for Additional file 1 and Additional file 2, respectively.

Additional file 2 further illustrates these concepts by showing GO term information for "GO: 0052248 modulation of programmed cell death in other organism during symbiotic interaction" and its child terms. Unlike the terms shown in Additional file 1, which reflect purely endogenous processes within a single organism, the terms included here are appropriate to use in describing genes in one organism whose products modulate programmed cell death in another organism, thus appropriately emphasizing the symbiotic interaction between different organisms. Indeed, these terms ultimately fall within the "GO: 0051704 multi-organism process" node of the GO biological process ontology (Figure 2; [1]), underscoring the notion of interaction between organisms.

The term "GO: 0052248 modulation of programmed cell death in other organism during symbiotic interaction" can be viewed (highlighted in black) in Figure 2, which depicts a greatly simplified directed acyclic graph (DAG; for more information on ontology structure see [13]) showing some more specific GO terms used to describe aspects of symbiont modulation of host programmed cell death. "GO: 0052040 modulation by symbiont of host programmed cell death" (shown in Figure 2, denoted by a dark star), or a child term of this more general parent term if more specific annotation information is available, would be used instead of "GO: 0012501 programmed cell death" (Additional file 1) to annotate any gene product produced by a symbiont that affected PCD in a host during a typical interaction. For example, the protein family, NPP1, comprises proteins from oomycetes, bacteria, and fungi that in plants cause HR-like cell death, pathogenesis-related gene transcription, reactive oxygen species (ROS) and ethylene (ET) generation, and apposition of callose, a (1→3)-β-d-glucan involved in both normal development and response to abiotic and biotic stress [31,32]. Annotating NPP1 family proteins with GO terms adds clarity not conferred by its literature description as a "necrosis-inducing protein". It would be appropriate to annotate a *Phytophthora sojae *member of the family (e.g. PsojNIP; [33]) with the GO term "GO: 0052040 modulation by symbiont of host programmed cell death" (Figure 2 and Additional file 2). Because an experiment showed that transient expression of PsojNIP in soybean tissue resulted in PCD [33], the annotation would be supported by a GO evidence code (IDA) that indicated a direct experimental assay was used (see the GO website for more information on evidence codes [34]).

When host defense is clearly implicated, for example when PCD is triggered by the detection of a pathogen MAMP by a host *R*-gene product, it would be appropriate to use the GO term "GO: 0034055 positive regulation by symbiont of host defense-related programmed cell death" (Figure 2). An example of this is a family of extracellular proteins called elicitins that are secreted by many *Phytophthora *species and that trigger localized cell death in *Nicotiana *host plant species [22]. The response of *Nicotiana benthamiana *to the elicitin INF1 prevents infection by *Phytophthora infestans *[35]. In this particular interaction, even though the triggering of PCD in the host is detrimental to the pathogen, it nevertheless reflects one action of the pathogen protein *in planta*. This underscores the notion that the purpose of GO terms is to describe biological processes, irrespective of whether the outcome of a process is subjectively judged to be beneficial or detrimental.

## Manipulation of PCD by diverse symbionts

Because PCD is a central mechanism of defense used by both animals and plants against microbes, manipulation by the symbiont of host PCD is central to many strategies by which symbionts neutralize host defenses. The following sections summarize some different strategies employed by symbionts for manipulation of host PCD. In these sections, we use the word "effector" to indicate symbiont gene products that influence the physiology or morphology of the host in order to promote colonization. Many effectors are proteins that modulate host defenses, including PCD (reviewed in [18,36,37]), and many of these are translocated into the cytoplasm of host cells [18,36,37]. In the context of plant defenses, most *R*-gene products detect symbiont effector proteins [18,36-38]. Historically, genes encoding effectors recognized by *R*-genes have been called "avirulence genes" [38].

### Viruses and PCD

In accord with the requirements of the different stages of viral replication in living cells, viruses both inhibit and induce apoptosis in host cells; this has been extensively studied in animal systems (reviewed in [39]). The suppression of host apoptosis by viruses is a critical aspect of prolonging cell survival during viral replication, which is captured in the GO by the term "GO: 0019050 suppression by virus of host apoptosis", a child term of "GO: 0052041 negative regulation by symbiont of host programmed cell death" (both shown in Figure 2) [1]. Suppression of the host immune response by inhibiting apoptosis is accomplished by viruses and viral proteins through targeting of host PCD signalling pathways [39].

As a normal part of the infection cycle of many viruses, the release and spread of progeny virions is accomplished by lysis of the host cell. To that end, diverse viruses and viral gene products facilitate induction of host cell apoptosis, a process that can be characterized by the GO term "GO: 0019051 induction by virus of host apoptosis" (Figure 2). Mechanisms to achieve this target many components of the host cell death signalling pathways (reviewed in [39]).

### Manipulation of PCD by bacterial pathogens of animals and plants

Bacterial pathogens of animals and plants can exert a pro-apoptotic effect on cells, or they can block apoptosis [40]. *Legionella pneumophila*, the Legionnaires' disease bacterium, induces host PCD as part of its pathogenic strategy through activation of the mitochondrial apoptosis pathway, including activation of caspases, BAX activation, and release of cytochrome *c *[41]. *Salmonella enterica *induces apoptosis in intestinal cells, but in macrophages it induces pyroptosis, a recently described caspase-1-dependent PCD pathway distinct from apoptosis [42], and for which a GO term has not yet been created. *Mycobacterium tuberculosis*, the causative agent of tuberculosis, induces macrophage apoptosis in humans by a tumour necrosis factor (TNF)-α-dependent mechanism. Induction of apoptosis by *M. tuberculosis *occurs in a strain-dependent manner [43], underscoring the variability of symbiont-host interactions. Annotating characterized proteins from *L. pneumophila*, *S. enterica*, or *M. tuberculosis *with "GO: 0052151 positive regulation by symbiont of host apoptosis" would facilitate useful comparison (Figure 2). In contrast, *Rickettsia rickettsii *can block apoptosis via activation of the transcription factor nuclear factor kappa B (NF-κB) pathway [40]. To describe blockage of host apoptosis, "GO: 0033668 negative regulation by symbiont of host apoptosis", a child of "GO: 0052150 modulation by symbiont of host apoptosis" (both shown in Figure 2), could be used.

Many bacterial pathogens of plants, including *Pseudomonas syringae *pathovars, *Ralstonia solanacearum, Xanthomonas *spp., and *Erwinia *spp., secrete effector proteins that can affect host cell defense signalling including the HR. Some are injected directly via type III or type IV secretion machinery into the host cell (reviewed in [44] and in this supplement [36,37,45]). Here, and in a following section describing necrotrophic fungi and bacteria, the roles of effectors in modulating PCD during *P. syringae *and *Pectobacterium carotovorum *(formerly *Erwinia carotovora*) infection are summarized briefly. Many effectors produced by *P. syringae *can either elicit or suppress the HR depending on the effector and *R*-gene repertoires of the interacting strains and plants [46-49], and thus *R*-gene mediated resistance is a practical approach to the protection of crops against *P. syringae *[50]. To annotate such effector proteins, one could use "GO: 0034053 modulation by symbiont of host defense-related programmed cell death", or either of its child terms, e.g. "GO: 0034054 negative regulation by symbiont of host defense-related programmed cell death" or "GO: 0034055 positive regulation by symbiont of host defense-related programmed cell death", depending on the context of the effector under consideration (Figure 2).

### Biotrophic pathogens and diverse mutualists suppress PCD

Biotrophic pathogens have evolved intricate mechanisms to colonize their hosts and maintain host cell integrity [51]. For example, intracellular pathogens, such as protozoan parasites and phytoplasmas (bacterial plant pathogens that lack cell walls), must thwart host defense responses while they derive nutrients from the host. If host PCD is triggered, an obligate biotroph must necessarily be destroyed. Suppression of host cell apoptosis is employed by many protozoans including: *Toxoplasma gondii*, an obligate parasite of mammals and birds; the Trypanosomatids *Trypanosoma cruzi*, which causes Chagas' disease, and *Leishmania donovani*, which causes visceral leishmaniasis; *Theileria parva *and *T. annulata*, tick-transmitted parasites of ruminant animals; *Plasmodium *species including the malaria parasites; and *Cryptosporidium parvum*, which causes cryptosporidiosis in mammals (all reviewed in [52]).

*Trypanosoma cruzi *appears to inhibit the Fas (CD95)-mediated cell death pathway; this pathway is triggered via TNF receptors and normally results in cytotoxic T cell activation [53]. *T. cruzi *suppressor proteins could be annotated with "GO: 0033668 negative regulation by symbiont of host apoptosis", thus facilitating comparison with functionally similar bacterial proteins. Interestingly, uninfected cells surrounding *Toxoplasma gondii*-infected cells undergo apoptosis, and recently a secreted molecule encoded by *T. gondii*, TgPDCD5, was shown to trigger PCD in these bystander cells [54], i.e. "GO: 0052042 positive regulation by symbiont of host programmed cell death" (Figure 2). Yet *T. gondii*-infected cells show a reduced response to many inducers of apoptosis, resulting from the blocking of several stages of the host mitochondrion-dependent PCD pathway [55], as well as direct inhibition of downstream caspase activation [55-57] and activation of NF-κB [58]. *Theileria parva *also appears to induce activation of NF-κB [59]. Thus, NF-κB activation may be a strategy used by diverse protozoan, viral and bacterial pathogens to inhibit apoptosis in the host [52], i.e. "GO: 0033668 negative regulation by symbiont of host apoptosis" (Figure 2).

In similar fashion, the effector protein ATR13 from the obligate biotrophic oomycete pathogen of *Arabidopsis*, *Hyaloperonospora arabidopsidis*, could suppress the ROS burst typically associated with immunity against the pathogen [60].

Mutualistic symbioses also involve manipulation of PCD. *Wolbachia *is an endosymbiotic bacterium that manipulates host reproduction in *Asobara tabida*, a parasitoid wasp. It accomplishes this by acting on host apoptotic pathways crucial to oogenesis, although the nature of control (host or symbiont) remains unclear [61]. In the fungal endophyte *Epichloe festucae*, generation of ROS has been shown to be a critical component of the mutualistic interaction with *Lolium perenne *(perennial ryegrass). Plants infected with fungal mutants lacking a functional NADPH oxidase, either through disruption of the gene *noxA *[62] or its small regulatory GTPase RacA [63], were stunted and lost apical dominance, most likely resulting from increased endophyte growth *in planta*. Although this was not due to localized host PCD [62], per se, it underscores the importance of ROS (often associated with PCD) in symbiotic interactions.

Gene products from organisms as diverse as the apicomplexan protozoon *Toxoplasma gondii*, the oomycete *Hyaloperonospora arabidopsidis*, the fungus *Epichloe festucae*, and the bacterium *Wolbachia *could have functional similarities revealed by GO annotation with "GO: 0052040 modulation by symbiont of host programmed cell death" (Figure 2 and Additional file 2).

### Necrotrophic fungi and bacteria promote PCD in plant hosts

In plants, as a generality, activation of salicylic acid-dependent pathways and PCD are the primary defense mechanisms against biotrophic pathogens, whereas jasmonic acid and ethylene signalling pathways mediate defense against necrotrophs [64], which are pathogens that gain their nutrition through host cell death. Consequently, biotrophs suppress host PCD, whereas necrotrophs actively facilitate host PCD [3,65]. Therefore, effective plant responses against necrotrophs often do not involve invoking HR-like PCD [66].

Some necrotrophic pathogens trigger host cell death by non-specific toxin production and ROS generation [67]. The HR and associated H_2_O_2 _were positively correlated in *Arabidopsis thaliana *with the growth of the necrotrophic fungus *Botrytis cinerea *[65]. Virulence-associated generation of H_2_O_2 _by *B. cinerea *is due, at least in part, to a Cu-Zn-superoxide dismutase BCSOD1; over-expression triggered H_2_O_2 _production and knockout mutants exhibited somewhat reduced virulence [68]. Another necrotrophic fungus, *Sclerotinia sclerotiorum*, secretes oxalic acid (OA), a non-host specific toxin [69] that may normally act as a signalling molecule in plants [70]. *S. sclerotiorum *showed greatly reduced disease symptoms on tomato plants expressing a wheat gene encoding oxalate oxidase [71], which detoxifies OA through conversion into CO_2 _and H_2_O_2 _[72]. Toxins that invoke PCD, or proteins responsible for synthesizing and exporting such toxins, would be annotated with "GO: 0052042 positive regulation by symbiont of host programmed cell death" (Figure 2).

Many necrotrophic phytopathogenic fungi and bacteria produce endopolygalacturonase (PG) enzymes that degrade cell wall pectin into oligogalacturonides and other products, and that may act directly to trigger PCD. During soybean infection, PGs from *S. sclerotiorum *could induce a sustained increase in intracellular Ca^2+^, leading to extracellular H_2_O_2 _accumulation and ultimately PCD [73]. Similarly, soft-rot enterobacteria, such as *Pectobacterium carotovorum*, secrete, via the type II secretion pathway, massive amounts of pectolytic enzymes, which can kill and macerate plant tissues, and they also possess a type III secretion system [74]. In the bryophyte *Physcomitrella patens*, *Pectobacterium carotovorum *could cause severe maceration of tissues and cytoplasmic shrinkage of protonemal cells, indicating host PCD despite induction of *PR-1 *(Pathogenesis-Related protein-1), *LOX *(lipoxygenase), *PAL *(phenylalanine ammonia-lyase), and *CHS *(chalcone synthase) defense genes [75]. Secretion of the HrpN harpin via the type III secretion system may promote this necrotroph-associated form of disease development [49]. The disease caused by *Pectobacterium carotovorum *on *Physcomitrella patens *closely resembles that caused by the necrotrophic fungus *Botrytis cinerea *[75]. The pectolytic enzymes in these pathogens could be described by "GO: 0052042 positive regulation by symbiont of host programmed cell death" (Figure 2) as well as "GO: 0052011 catabolism by symbiont of host cell wall pectin".

### Hemibiotrophic fungal and oomycete pathogens

Hemibiotrophic plant pathogens initially suppress or avoid triggering PCD during the biotrophic phase of infection, but then actively promote cell death during the transition to necrotrophy [33]. The mechanism(s) underlying the switch from biotrophy to necrotrophy remain largely unknown [2]. In *P. sojae*, expression of the protein toxin PsojNIP is associated with the transition to necrotrophy, and has been hypothesized to be responsible for the switch [33]. In wheat infected with the host-specific fungal pathogen *Mycosphaerella graminicola*, disease symptoms often do not appear for several weeks. Once the necrotrophic stage begins, however, the host exhibits PCD-like characteristics, along with increased cell membrane leakage and apoplastic metabolite levels, which correlate with increased fungal growth, membrane transport, and metabolism [76]. A similar situation exists in *Fusarium graminearum*, which lives biotrophically before switching to necrotrophy; following exposure to *F. graminearum*-derived trichothecene mycotoxins, multiple barley transcripts were detected including a PCD-related pirin [77], which may signify pathogen-triggered PCD.

The effector Avr3a of *Phytophthora infestans*, expressed during early infection of potato, can suppress the PCD triggered by the MAMP elicitin [78], i.e. "GO: 0034054 negative regulation by symbiont of host defense-related programmed cell death" (Figure 2). Similarly, several effectors from *P. sojae*, including Avr1b, could suppress BAX-triggered PCD, and were hypothesized to have a physiological role of suppressing defense-associated PCD [79]. *P. infestans *Avr3a and *P. sojae *Avr1b also can be described with "GO: 0034055 positive regulation by symbiont of host defense-related programmed cell death" (Figure 2) as they trigger the host HR when the host resistance genes *R*3a or *Rps*1b, respectively, are present [78,79], which underscores the complex roles of effectors and the need for careful annotation of them.

## Conclusion

Plants and animals share many similarities with respect to immunity and defense [80], and symbionts employ a wide diversity of mechanisms to modulate these defenses. Diverse symbionts, ranging from pathogenic to mutualistic, have evolved mechanisms for influencing host programmed cell death to neutralize host defenses, expand the area and duration of host colonization, and improve survival. The PAMGO Consortium, to describe processes involved in host-microbe interactions, has created a large number of Gene Ontology terms, including a set of terms to describe PCD in the context of host-symbiont interactions. The manipulation of PCD by diverse symbionts is a complex and rapidly evolving research area. The more that these terms are used, refined and added to by the community, the more that they will enhance our ability to identify common mechanisms by which symbionts influence death processes occurring within their hosts.

## Note added in proof

A recent report from the Nomenclature Committee on Cell Death [81] has noted that in some cases necrosis may result from an orderly process, but great caution still needs to be applied in the use of the term.

## List of abbreviations used

DAG: directed acyclic graph; ET: ethylene; GO: Gene Ontology; HR: hypersensitive response; MAMP: microbe-associated molecular pattern; NF-κB: nuclear factor kappa B; NPP1: necrosis inducing *Phytophthora *protein; OA: oxalic acid; PAMGO: Plant-Associated Microbe Gene Ontology; PCD: programmed cell death; PG: endopolygalacturonase; *R*-gene: resistance gene; ROS: reactive oxygen species;  TNF: tumour necrosis factor.

## Competing interests

The authors declare that they have no competing interests.

## Authors' contributions

MCC wrote the manuscript based on discussions with the other co-authors, who also edited the manuscript. All authors contributed to the development of Gene Ontology terms describing programmed cell death.

## Supplementary Material

Additional file 1 **Selected commonly used terms related to endogenous cell death, as defined by the Gene Ontology**. The GO terms described here refer to endogenous processes found in the biological process ontology. "Concept" refers to the term as commonly found in the literature. This word or phrase was queried against the Gene Ontology using the search function in AmiGO, the GO browser [1]. The other rows ("Term name", "Accession", "Synonyms", "Definition", and "Comment") represent fields from the term information for selected GO terms resulting from the query. In the case of "necrosis", no specific GO term exists (and thus the "Comment" field is an author comment), but "necrosis" exists as a synonym to several GO terms (but see [81]). Three of the terms shown here suggest (in the comment) alternative terms that can be used for annotating PCD in host-symbiont interactions; these alternative terms can be found in Figure 2, highlighted with broken lines. All GO terms below exist in the biological process ontology. For brevity, several other PCD-related GO terms are not shown: "GO: 0048102 autophagic cell death", "GO: 0016244 non-apoptotic programmed cell death", "GO: 0010623 developmental programmed cell death", "GO: 0043067 regulation of programmed cell death", "GO: 0043069 negative regulation of programmed cell death", "GO: 0043068 positive regulation of programmed cell death", and "GO: 0010343 singlet oxygen-mediated programmed cell death". Click here for file

Additional file 2 **"GO: 0052248 modulation of programmed cell death in other organism during symbiotic interaction" and child terms**. Selected term information fields ("Term name", "Accession", "Synonyms", and "Definition") are shown for each GO term. Unlike the terms shown in Table 1, the terms included here are appropriate to use in describing genes in one organism whose products modulate programmed cell death in another organism. For more context, "GO: 0052248 modulation of programmed cell death in other organism during symbiotic interaction" can be seen also in Figure 2, highlighted in black. Click here for file
