# Supplementary material for: Programmed cell death in host-symbiont associations, viewed through the Gene Ontology
Source: BMC Microbiol. 2009 Feb 19;9(Suppl 1):S5. doi: 10.1186/1471-2180-9-S1-S5 (PMC2654665; doi:10.1186/1471-2180-9-S1-S5)
Supplement: Additional file 1 — Selected commonly used terms related to endogenous cell death, as defined by the Gene Ontology. The GO terms described here refer to endogenous processes found in the biological process ontology. "Concept" refers to the term as commonly found in the literature. This word or phrase was queried against the Gene Ontology using the search function in AmiGO, the GO browser [1]. The other rows ("Term name", "Accession", "Synonyms", "Definition", and "Comment") represent fields from the term information for selected GO terms resulting from the query. In the case of "necrosis", no specific GO term exists (and thus the "Comment" field is an author comment), but "necrosis" exists as a synonym to several GO terms (but see [81]). Three of the terms shown here suggest (in the comment) alternative terms that can be used for annotating PCD in host-symbiont interactions; these alternative terms can be found in Figure 2, highlighted with broken lines. All GO terms below exist in the biological process ontology. For brevity, several other PCD-related GO terms are not shown: "GO: 0048102 autophagic cell death", "GO: 0016244 non-apoptotic programmed cell death", "GO: 0010623 developmental programmed cell death", "GO: 0043067 regulation of programmed cell death", "GO: 0043069 negative regulation of programmed cell death", "GO: 0043068 positive regulation of programmed cell death", and "GO: 0010343 singlet oxygen-mediated programmed cell death". [file 1471-2180-9-S1-S5-S1.doc]

**Additional file 1**

Selected commonly used terms related to endogenous cell death, as defined by the Gene Ontology.

| Concept | Programmed cell death |
| --- | --- |
| **Term name** | **Programmed cell death** |
| Accession | GO : 0012501 |
| Synonyms | Related: necrosis |
| Definition | Cell death resulting from activation of endogenous cellular processes. |
| Comment | Note that this term should be used to annotate gene products in the organism undergoing the programmed cell death. To annotate genes in another organism whose products modulate programmed cell death in a host organism, **consider the term “modulation by symbiont of host programmed cell death; GO:0052040”**. |
|  | |
| Concept | Programmed cell death |
| **Term name** | **Host programmed cell death induced by symbiont** |
| Accession | GO : 0034050 |
| Synonyms | None |
| Definition | Cell death in a host resulting from activation of host endogenous cellular processes after direct or indirect interaction with a symbiont (defined as the smaller of two, or more, organisms engaged in symbiosis, a close interaction encompassing mutualism through parasitism). An example of direct interaction is contact with penetrating hyphae of a fungus; an example of indirect interaction is encountering symbiont-secreted molecules. |
| Comment | Note that this term is to be used to annotate gene products in the host, not the symbiont. To annotate gene products in the symbiont that induce programmed cell death in the host, **consider the biological process term “induction by symbiont of host programmed cell death; GO:0052044”**. |
|  | |
| Concept | Hypersensitive response |
| **Term name** | **Plant-type hypersensitive response** |
| Accession | GO : 0009626 |
| Synonyms | Exact: HR  Exact: HR-PCD  Exact: plant hypersensitive response |
| Definition | The rapid, localized death of plant cells in response to invasion by a pathogen. |
| Comment | Note that term is to be used to annotate gene products in the plant. To annotate symbiont gene products that induce the hypersensitive response, **consider the biological process term “modulation by symbiont of host defense-related programmed cell death; GO:0034053”**. |
|  | |
| **Concept** | **Necrosis** |
| Synonyms | Query matches synonym "necrosis" [related synonym] for the following terms:  GO : 0008219 cell death  GO : 0001906 cell killing  GO : 0019835 cytolysis  GO : 0012501 programmed cell death |
| Comment | “Necrosis” describes a phenotype and not a process. Its usage is also highly varied in the literature, especially between plant and animal systems. Its usage as a synonym for “cell death” is discouraged. |
|  | |
| Concept | Apoptosis |
| **Term name** | **Apoptosis** |
| Accession | GO : 0006915 |
| Synonyms | Related: signaling (initiator) caspase activity  Narrow: type I programmed cell death  Exact: apoptotic programmed cell death  Exact: programmed cell death by apoptosis |
| Definition | A form of programmed cell death induced by external or internal signals that trigger the activity of proteolytic caspases, whose actions dismantle the cell and result in cell death. Apoptosis begins internally with condensation and subsequent fragmentation of the cell nucleus (blebbing) while the plasma membrane remains intact. Other characteristics of apoptosis include DNA fragmentation and the exposure of phosphatidyl serine on the cell surface. |
| Comment | None |
|  | |
| Concept | Programmed cell death |
| **Term name** | **Hydrogen peroxide-mediated programmed cell death** |
| Accession | GO : 0010421 |
| Synonyms | None |
| Definition | Programmed cell death induced by hydrogen peroxide. Programmed cell death is the cell death resulting from activation of endogenous cellular processes. |
| Comment | None |

The GO terms described here refer to endogenous processes found in the biological process ontology. “Concept” refers to the term as commonly found in the literature. This word or phrase was queried against the Gene Ontology using the search function in AmiGO, the GO browser [1]. The other rows (“Term name”, “Accession”, “Synonyms”, “Definition”, and “Comment”) represent fields from the term information for selected GO terms resulting from the query. In the case of “necrosis,” no specific GO term exists (and thus the “Comment” field is an author comment), but “necrosis” exists as a synonym to several GO terms. Three of the terms shown here suggest (in the comment) alternative terms that can be used for annotating PCD in host-symbiont interactions; these alternative terms can be found in Figure 2, highlighted with broken lines. All GO terms below exist in the biological process ontology. For brevity, several other PCD-related GO terms are not shown: “GO : 0048102 autophagic cell death”, “GO : 0016244 non-apoptotic programmed cell death”, “GO : 0010623 developmental programmed cell death”, “GO : 0043067 regulation of programmed cell death”, “GO : 0043069 negative regulation of programmed cell death”, “GO : 0043068 positive regulation of programmed cell death”, and “GO : 0010343 singlet oxygen-mediated programmed cell death”.
