# Supplementary material for: Programmed cell death in host-symbiont associations, viewed through the Gene Ontology
Source: BMC Microbiol. 2009 Feb 19;9(Suppl 1):S5. doi: 10.1186/1471-2180-9-S1-S5 (PMC2654665; doi:10.1186/1471-2180-9-S1-S5)
Supplement: Additional file 2 — "GO: 0052248 modulation of programmed cell death in other organism during symbiotic interaction" and child terms. Selected term information fields ("Term name", "Accession", "Synonyms", and "Definition") are shown for each GO term. Unlike the terms shown in Table 1, the terms included here are appropriate to use in describing genes in one organism whose products modulate programmed cell death in another organism. For more context, "GO: 0052248 modulation of programmed cell death in other organism during symbiotic interaction" can be seen also in Figure 2, highlighted in black. [file 1471-2180-9-S1-S5-S2.doc]

**Additional file 2**

**“GO : 0052248 modulation of programmed cell death in other organism during symbiotic interaction” and child terms.**

| **Term name** | **Modulation of programmed cell death in other organism during symbiotic interaction** |
| --- | --- |
| Accession | GO : 0052248 |
| Synonyms | None |
| Definition | Any process by which an organism modulates the frequency, rate or extent of programmed cell death in a second organism, where the two organisms are in a symbiotic interaction. |
|  | |
| **Term name** | **Modulation by organism of apoptosis in other organism during symbiotic interaction** |
| Accession | GO : 0052433 |
| Synonyms | Exact: modulation by organism of apoptotic programmed cell death in other organism during symbiotic interaction |
| Definition | Any process by which an organism modulates the frequency, rate or extent of programmed cell death by apoptosis in a second organism, where the two organisms are in a symbiotic interaction. |
|  | |
| **Term name** | **Modulation by organism of non-apoptotic programmed cell death in other organism during symbiotic interaction** |
| Accession | GO : 0052459 |
| Synonyms | None |
| Definition | Any process by which an organism modulates the frequency, rate or extent of programmed cell death by a non-apoptotic pathway in a second organism, where the two organisms are in a symbiotic interaction. |
|  | |
| **Term name** | **Modulation by symbiont of host programmed cell death** |
| Accession | GO : 0052040 |
| Synonyms | Exact: modulation of host PCD |
| Definition | Any process by which an organism modulates the frequency, rate or extent of programmed cell death in the host organism. The host is defined as the larger of the organisms involved in a symbiotic interaction. |
|  | |
| **Term name** | **Negative regulation by organism of programmed cell death in other organism during symbiotic interaction** |
| Accession | GO : 0052490 |
| Synonyms | Narrow: inhibition by organism of programmed cell death in other organism during symbiotic interaction  Exact: down regulation by organism of programmed cell death in other organism during symbiotic interaction  Exact: down-regulation by organism of programmed cell death in other organism during symbiotic interaction  Exact: downregulation by organism of programmed cell death in other organism during symbiotic interaction  Exact: inhibition of programmed cell death in other organism |
| Definition | Any process by which an organism stops, prevents or reduces the frequency, rate or extent of programmed cell death in a second organism, where the two organisms are in a symbiotic interaction. |
|  | |
| **Term name** | **Positive regulation by organism of programmed cell death in other organism during symbiotic interaction** |
| Accession | GO : 0052330 |
| Synonyms | Narrow: activation by organism of programmed cell death in other organism during symbiotic interaction  Narrow: stimulation by organism of programmed cell death in other organism during symbiotic interaction |
| Definition | Any process by which an organism activates, maintains or increases the frequency, rate or extent of programmed cell death in a second organism, where the two organisms are in a symbiotic interaction. |

Selected term information fields (“Term name”, “Accession”, “Synonyms”, and “Definition”) are shown for each GO term. Unlike the terms shown in Table 1, the terms included here are appropriate to use in describing genes in one organism whose products modulate programmed cell death in another organism. For more context, “GO : 0052248 modulation of programmed cell death in other organism during symbiotic interaction” can be seen also in Figure 2, highlighted in black.
